# Five centuries of genome evolution and multi-host adaptation of Campylobacter jejuni in Brazil

**DOI:** 10.1099/mgen.0.001274

**Published:** 2024-07-19

**Authors:** Ana Beatriz Garcez Buiatte, Stephanie S. R. Souza, Leticia Roberta Martins Costa, Phelipe Augusto Borba Martins Peres, Roberta Torres de Melo, Simone Sommerfeld, Belchiolina Beatriz Fonseca, Nicole I. Zac Soligno, Odion O. Ikhimiukor, Paulo Marcel Armendaris, Cheryl P. Andam, Daise Aparecida Rossi

**Affiliations:** 1 Molecular Epidemiology Laboratory, Federal University of Uberlândia, Uberlândia, Minas Gerais, Brazil; 2 Department of Biological Sciences, University at Albany, State University of New York, Albany, New York, USA; 3 Nanobiotechnology Laboratory, Federal University of Uberlândia, Uberlândia, Minas Gerais, Brazil; 4 Infectious Disease Laboratory, Federal University of Uberlândia, Uberlândia, Minas Gerais, Brazil; 5 Federal Agriculture Defense Laboratory/RS - LFDA/RS, Porto Alegre, Rio Grande do Sul, Brazil

**Keywords:** antimicrobial resistance, *Campylobacter jejuni*, genome evolution, population structure, sequence type 353, virulence

## Abstract

Consumption of raw, undercooked or contaminated animal food products is a frequent cause of *Campylobacter jejuni* infection. Brazil is the world’s third largest producer and a major exporter of chicken meat, yet population-level genomic investigations of *C. jejuni* in the country remain scarce. Analysis of 221 *C*. *jejuni* genomes from Brazil shows that the overall core and accessory genomic features of *C. jejuni* are influenced by the identity of the human or animal source. Of the 60 sequence types detected, ST353 is the most prevalent and consists of samples from chicken and human sources. Notably, we identified the presence of diverse *bla* genes from the OXA-61 and OXA-184 families that confer beta-lactam resistance as well as the operon *cmeABCR* related to multidrug efflux pump, which contributes to resistance against tetracyclines, macrolides and quinolones. Based on limited data, we estimated the most recent common ancestor of ST353 to the late 1500s, coinciding with the time the Portuguese first arrived in Brazil and introduced domesticated chickens into the country. We identified at least two instances of ancestral chicken-to-human infections in ST353. The evolution of *C. jejuni* in Brazil was driven by the confluence of clinically relevant genetic elements, multi-host adaptation and clonal population growth that coincided with major socio-economic changes in poultry farming.

**Impact Statement**

Ingestion of food products contaminated with *Campylobacter jejuni* is the major cause of foodborne illnesses worldwide. Our genomic surveillance work shows that the evolution of *C. jejuni* in Brazil was driven by the convergence of clinically relevant genetic elements, multi-host adaptation and rapid clonal expansion likely attributed to major socio-economic changes in poultry farming. Restricting the spread of pathogenic and drug-resistant *C. jejuni* in poultry populations should, therefore, be a priority to limit its impact on food security and public health, especially in poorly surveyed countries such as Brazil. Our findings will inform public health efforts to reduce the burden of campylobacteriosis, including the use of effective antimicrobials, control of zoonosis, design of food safety measures, source traceback and outbreak detection.

## Data Summary

Genome sequence data of the 105 *Campylobacter jejuni* isolates sequenced in this study are available in the National Center for Biotechnology Information Sequence Read Archive database under BioProject accession number PRJNA1043437. BioSample accession numbers for all genomes are listed in Table S1, available in the online Supplementary Material. The authors confirm that all supporting data and protocols have been provided within the article or through supplementary data files.

## Introduction

*Campylobacter* is the most frequently detected bacterial pathogen in foodborne gastroenteritis cases in countries that have active epidemiological surveillance [1,2]. Worldwide, the incidence of campylobacteriosis varies by country and ranges from 11 to 1512 per 100 000 population per year [1], thus representing a major economic and public health burden. *Campylobacter jejuni* is responsible for approximately 90 % of campylobacteriosis cases [2]. *C. jejuni* is a part of the normal intestinal microbiota of many animals [3], and hence, human infection is often associated with the consumption of raw, undercooked or contaminated animal food products [4]. Poultry meat is most frequently associated with *C. jejuni* disease transmission and outbreaks [4,5]. Affected humans develop gastrointestinal symptoms such as diarrhoea, abdominal pain, fever, nausea and vomiting, which usually resolve with only supportive care [6]. However, in more severe cases and especially in individuals with acute or persistent infections, weakened immune system or severe comorbidities, antimicrobials are commonly prescribed [1]. Macrolides and fluoroquinolones are the drugs of choice for the treatment of *C. jejuni* infections [7], although resistance to these antimicrobials has been increasing [8]. *Campylobacter* infection is the most commonly identified cause of the rare neurological disorder Guillain–Barré syndrome (GBS), whereby a person’s immune system attacks the peripheral nerves and causes muscle weakness and sometimes paralysis [9]. It is estimated that 5–41 % of GBS cases are associated with previous *Campylobacter* infection [10].

Whole-genome sequencing has been widely used in *Campylobacter* investigations, particularly as an integral part of surveillance efforts and outbreak detection [11], source attribution [5,12], discovery of new drug targets [13] and tracking of the spread of multidrug-resistant strains [14]. Genome sequences offer critical insights into understanding the ability of *Campylobacter* to colonize, infect and adapt to both human and animal hosts [15]. Nonetheless, this technology is not yet accessible to all countries, and many low- and medium-income countries have limited capability and resources to carry out high-throughput sequencing and bioinformatics. The lack of adequate diagnostic methods further underestimates the incidence and prevalence of campylobacteriosis in many countries [1,2]. In South America, limited genomic and epidemiological data on *C. jejuni* hinder a comprehensive understanding of the history and dynamics of *C. jejuni* infection, transmission and spread of antimicrobial resistance (AMR). This is particularly evident in Brazil, where a structured surveillance programme for *Campylobacter* is lacking, despite it being the world’s largest exporter of chicken meat and having high domestic consumption of chicken products [16,17]. Brazil has recorded only 11 cases of campylobacteriosis between 2000 and 2021 [18]. From May to July 2019, there were 683 suspected or confirmed GBS cases in Peru, which was well in excess of the 1.2 cases/100 000 persons/year (i.e. 29 cases/year) [19]. The 2019 GBS outbreak in Peru was associated with *C. jejuni* sequence type (ST) 2993, which had been circulating in Peruvian Amazonia since 2003 [20]. Since this outbreak, *C. jejuni*-associated GBS cases in Peru remain exceptionally high from 2020 to 2023 [21]. In the Peruvian Amazon, *C. jejuni* from asymptomatic paediatric infection was associated with phylogenetically diverse but globally circulating lineages, with poultry as the most likely source of infection [22]. In Chile, high ciprofloxacin resistance was reported in patients with acute gastroenteritis between 2006 and 2015, mainly from ST21, ST48 and ST353 [23]. In Ecuador, 77.8 % of * C. jejuni* isolates sampled from healthy well-nourished children were resistant to nalidixic acid and ciprofloxacin [24].

Here, we aim to characterize the population genomic structure and evolutionary history of *C. jejuni* in Brazil. Analysis of 221 genomes from diverse geographical locations and ecological sources sampled between 1996 and 2018 revealed the growing importance of ST353, particularly in terms of its multidrug resistance, virulence and chicken-to-human infections. The diversification and expansion of ST353 began in the late 1500s and was likely facilitated by large socio-economic changes in Brazil that caused a rapid growth in intensive poultry farming. Our study highlights the need for continued genomic surveillance of *C. jejuni* in both clinical and agricultural settings to control the public health threat of *C. jejuni* infections. Crucially, it provides profound insights into how anthropogenic activities can shape the evolutionary trajectory and favour the expansion of certain high-risk clones.

## Methods

### Bacterial samples

We collected 105 *C*. *jejuni* isolates isolated from chicken carcasses slaughtered between 2011 and 2018 from six Brazilian states [Minas Gerais (MG), São Paulo (SP), Rio de Janeiro (RJ), Paraná (PR), Santa Catarina (SC) and Rio Grande do Sul (RS)] and Distrito Federal (DF) where the federal capital of Brazil (Brasília) is located (Table S1). These isolates came from slaughterhouses under federal inspection by the official veterinary service of the Ministry of Agriculture, Livestock and Food Supply and have been previously described in references [25,26]. Bacteria were grown in *Campylobacter* blood-free selective agar (modified charcoal-cefoperazone-deoxycholate agar-Preston) (CM0739, Oxoid) incubated at 37 °C for 44 h ± 4 h in a microaerobic atmosphere (5–15 % O_2_ and 10 % CO_2_) using a Microaerobac (Probac do Brazil, SP, Brazil). All isolates were stored in skim milk at −80 °C in the library of the Molecular Epidemiology Laboratory of the Department of Veterinary Medicine at the Federal University of Uberlândia.

### DNA extraction, library preparation and whole-genome sequencing

DNA was extracted using the Zymo Research Quick-DNA Fungal/Bacterial Miniprep Kit (Irvine, California, USA) following the manufacturer’s protocol. DNA concentration was measured using Qubit fluorometer (Invitrogen, Grand Island, New York, USA). Whole-genome sequencing was carried out at SeqCoast Genomics (Portsmouth, New Hampshire, USA). Samples were prepared for whole-genome sequencing using the Illumina DNA Prep tagmentation kit and unique dual indexes. Sequencing was performed on the Illumina NextSeq2000 platform using a 300-cycle flow cell kit to produce 2×150 bp paired reads. A 1–2 % PhiX control was spiked into the run to support optimal base calling. Read demultiplexing, read trimming and run analytics were performed using DRAGEN v.3.10.12 installed on the NextSeq2000.

### *De novo* genome assembly, sequence quality check and annotation

We used Shovill v.1.1.0 (https://github.com/tseemann/shovill) to assemble the paired-end read sequences into contigs. We used the –trim option to remove the adapter sequences. Shovill implements subsampling at a depth of 150×, read error connection, correcting sequencing and assembly errors and assembly using SPAdes [27]. To place the 105 genomes in a wider geographical context, we retrieved raw reads and assemblies of all *C. jejuni* genomes from Brazil that were available in the National Center for Biotechnology Information (NCBI) Sequence Read Archive (SRA) database (120 genomes as of May 2023). The *C. jejuni* genomes from NCBI were derived from humans, chicken, monkeys and sewage sources sampled between 1996 and 2018 from four states (MG, SP, RJ and RS) (Table S1). These were also assembled using Shovill.

To assess the quality of the genomes we sequenced and the publicly available genomes, we used QUAST v.5.0.2 [28] and CheckM v.1.1.3 [29]. We calculated the genome completeness (mean=99.96 %, range: 99.58–100.00 %) and genome contamination (mean=0.03 %, range=0.00–1.43 %) (Table S1), which were all within the genome quality standards recommended by CheckM [29]. Genomes with <90 % completeness and >5 % contamination were excluded. We excluded assemblies with >200 contigs and an N50 <40 000 bp. The number of contigs ranged from 11 to 96 (median = 31.22) and N50 between 102 961 and 1 039 954 bp (median = 200 286.63 bp) (Table S1). Altogether, our dataset included 105 genomes sequenced in this study and 116 from the NCBI, for a total of 221 genomes used in all downstream analyses. The assembled draft genomes were annotated using Prokka v.1.14.6 [30].

### Species validation and pan-genome calculation

To confirm that the genomes belong to the same species, we calculated the genome-wide average nucleotide identity (ANI) for all possible pairs of genomes using FastANI v.1.32 [31] (Table S2). ANI refers to the mean nucleotide identity of all orthologous pairs of genes that are shared between a pair of genomes [31]. We used the recommended ≥95 % ANI threshold to delineate species boundaries [31]. We compared the 105 genomes with the reference genome *C. jejuni* NCTC 11168 (GenBank accession number GCA_000009085.1). The annotated genomes were used as input to characterize the pan-genome, i.e. the totality of genes of all strains in our dataset [32], using Panaroo v.1.3.3 [33] (Table S3). Panaroo uses a graph-based gene clustering tool that is capable of detecting errors in annotations and filtering sequence contaminations. To ensure that only high-quality sequences are included in clusters, we used the -strict option. We defined core genes as those present in ≥99 % of the genomes, whereas the accessory genes were those present in >1 % and <99 % of the genomes. Nucleotide sequences of individual gene families were aligned using Prank [34].

### Phylogenetic reconstruction and population structure analysis

Nucleotide sequence alignments of 1412 core genes from the 221 genomes were concatenated to generate the core genome alignment. We used SNP-sites v.2.5.1 [35] to extract single nucleotide polymorphisms (SNP) from the core genome alignment generated by Panaroo. Using the core genome SNP alignment as input, we built a maximum likelihood phylogenetic tree using the program RAxML v.8.2.12 [36] with a general time reversible [37] model of nucleotide substitution and gamma distribution of rate heterogeneity. The phylogenetic tree was visualized using the online platform Interactive Tree of Life [38].

Using the core genome alignment as input, we partitioned the genomes into sequence clusters (SCs) of genetically similar individuals using the Bayesian hierarchical clustering algorithm fast Bayesian Analysis of Population Structure (fastBAPS v.1.0.8) [39]. For every pair of genomes, we calculated the genetic distance based on the core genome SNPs using SNP-dist v.0.8.2 (https://github.com/tseemann/snp-dists). We also calculated the core SNP distance only among the human isolates, among only the chicken isolates and between chicken and human isolates, based on the core genome alignment of each group.

### Comparison of *C. jejuni* population isolates in South America

To place the Brazilian *C. jejuni* in the broader South American context, we obtained 374 raw reads and assemblies of *C. jejuni* from NCBI (as of February 2024). These genomes came from Chile, Ecuador, Paraguay and Peru and were sourced from chickens, humans and dogs spanning 2010 to 2022 (Table S4). We conducted genome assembly, quality assessment, annotation, pangenome analysis, phylogenetic reconstruction and ST determination using the same methodologies employed for the Brazilian genomes. Nucleotide sequence alignments of 1377 core genes from the 595 genomes (i.e. 221 from Brazil and 374 from other South American countries) were concatenated to generate the core genome alignment.

### *In silico* identification of ST, AMR and virulence genes

We determined the ST of each isolate using multi-locus sequence typing (MLST) v.2.19.0 (https://github.com/tseemann/mlst), which extracts seven single-copy housekeeping genes (*aspA*, *glnA*, *gltA*, *glyA*, *pgm*, *tkt* and *uncA*) [40] and compares their sequence identity to previously deposited allele combinations in the *C. jejuni* database of pubMLST [41] (https://pubmlst.org/organisms/campylobacter-jejunicoli). Novel ST designations were assigned by pubMLST. We used ABRicate v.1.0.0 (https://github.com/tseemann/abricate) and AMRFinderPlus [42] to determine the presence of acquired AMR and virulence genes in using threshold values of >80 % sequence identity and >80 % sequence coverage. We compared the AMR genes related to efflux pump and virulence genes from the Brazilian isolates to those found in the Comprehensive Antibiotic Resistance Database [43] and the Virulence Factor Database [44]. We also created a custom database in ABRicate v.1.0.0 to detect the virulence genes *sodB*, *dnaJ*, *luxS*, *hcp*, *pldA* and *htrA*, using the threshold values of >80 % sequence identity and coverage. The AMR and virulence genes identified for every genome are listed in Table S5. The accession numbers for the genes used to construct the custom database are listed in Table S6.

### Time-calibrated phylogeny and population demographic analysis

For ST353, we generated recombination-free sequence alignment of concatenated core genes using Gubbins v.3.2.1 [45]. We first tested the presence of a temporal signal in our dataset. We used the recombination-free alignment as input in the root-to-tip function in BactDating v.1.1.1 [46] to carry out a root-to-tip linear regression analysis and random permutation of sampling dates to assess significance. BactDating uses the root-to-tip linear regression to provide a default starting point for the Markov Chain Monte Carlo (MCMC) algorithm to estimate the node ages in a time-calibrated phylogeny [46]. To obtain the time to the most recent common ancestor (tMRCA), we used BactDating with an additive relaxed clock model [47] and 10^8^ iterations to build a time-calibrated phylogeny. The first half of iterations (burn-in) was removed, and sampling was conducted every 1000 iterations. To evaluate the significance of the molecular clock signal, we ran BactDating with all sampling dates set uniformly and using the same model and number of iterations as in the original run. Subsequently, we employed the modelcompare function of BactDating to compute the deviance information criterion (DIC) between the two runs. The DIC is a metric utilized to assess the fit and complexity of Bayesian models [48]. The year 2004 was assigned to all samples in this second run, which was derived from the randomization between the years 1996 and 2018 using the lubridate v.1.9.292 [49] package in R v.2023.06.1 [44]. We used the coda v.0.19-4.1 package [50] to assess the convergence and mixing of the MCMC chains. The effective sample sizes of the inferred parameters *α*, *μ* and *σ* were computed to ensure they exceeded the recommended threshold of 200. Moreover, we carried out an additional BactDating run employing sample dates identical to the original run and the same model and number of iterations and compared with the original run to ensure that the multivariate version of the Gelman–Rubin statistic was below the recommended threshold of 1.1 [51] (Table S7). The Gelman–Rubin statistic is used for monitoring the convergence of iterative simulations by comparing between and within variances of multiple chains [52].

To investigate potential clonal expansions in the ST353 population, we employed the CaveDive v.0.1.1 package [53] with the dated phylogenetic tree output from BactDating as an input. The MCMC sampler was carried out for 10^8^ iterations with sampling every 1000 iterations. We also used the coda v.0.19-4.1 package [50] to evaluate the convergence and mixing properties of our MCMC sampler. We found these properties to be satisfactory, with Gelman–Rubin statistics below 1.1 and effective sample sizes exceeding 200 for all parameters (Table S8).

To estimate the effective population size *Ne*, we used Skygrowth v.0.3.1 [54], which performs a nonparametric phylodynamic inference using maximum *a posteriori* and MCMC methods for model fitting. *Ne* refers to the rate of change in population composition due to genetic drift [55].

### Statistical analysis

We carried out all statistical analyses using the ggstastsplot v.0.12.0 [56] package in RStudio v.2023.06.1 [57]. We used Spearman’s rank correlation coefficient to estimate the number of virulence and AMR genes as a function of time (years). We used Mann–Whitney’s test to compare the genomes from human and chicken sources in terms of genome size and the number of accessory genes. We used the Kruskal–Wallis test to compare the number of virulence and AMR genes per source and the ANI and core SNP distances of genomes from chicken, human and both sources. Results were considered significant when *P* < 0.05.

## Results

### Population structure of *C. jejuni* in Brazil

**Fig. 1.**
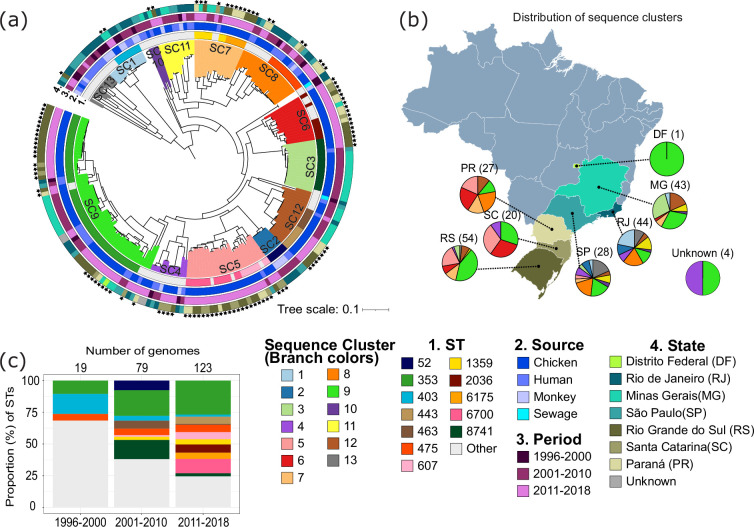
Phylogenetic, geographical and ecological distribution of 221 *C. jejuni* genomes from Brazil. (**a**) Midpoint-rooted maximum likelihood phylogenetic tree built from the sequence alignment of 1412 core genes. Tree scale represents the number of nucleotide substitutions per site. The black stars indicate the genomes that were sequenced in this study. Outer rings show the (1) ST, (2) source of isolation, (3) year of sampling and (4) sampling site. Branches are coloured according to BAPS sequence clusters (SCs). (**b**) Distribution of BAPS sequence clusters in each Brazilian state. Four genomes do not have geographical information and are labelled unknown. The colours in the pie charts indicate the BAPS sequence cluster. The numbers above each pie chart represent the number of genomes from each state. (**c**) Proportion and number of *C. jejuni* genomes from different STs classified according to the period of collection (categorized by decade). The total number of genomes per period is shown at the top of the bars.

Our Brazilian *C. jejuni* dataset consisted of 105 isolates from chicken carcasses that we sequenced, augmented with 116 genomes retrieved from the NCBI SRA database that were sampled from any location in Brazil and from any ecological source. In all, 221 high-quality genome sequences representing samples from six states (MG, SP, RJ, PR, SC and RS) and DF recovered between 1996 and 2018 were included in all downstream analyses (Fig. 1a–c and Table S1). The dataset included genomes derived from chicken (152/221 or 68.78 % of the population), human (48/221, 21.72 %), monkey (19/221, 8.60 %) and sewage (2/221, 0.90 %). Four genomes do not have information about the states where they were collected.

*De novo* genome assembly generated sequences of sizes ranging from 1.59 to 1.90 Mb (mean=1.72 Mb) Table S1 and Fig. S1a). The totality of genes in the population or pan-genome [32] consisted of 2944 orthologous gene families (Table S3). A total of 1412 genes comprised the core genome (i.e. genes present in ≥ 99 % genomes), which represented approximately 48 % of the pan-genome. The number of accessory genes per genome ranged from 156 to 528 (mean=316) (Fig. S1b). A total of 190 accessory genes (representing 6.45 % of the pan-genome) were present only in single genomes. The genome-wide ANI [31] values of each genome compared to the reference genome and between every possible pair of genomes in our dataset were ≥96 % (Table S2 and Fig. S1c).

We constructed a maximum likelihood tree based on 59 483 SNPs extracted from the nucleotide sequence alignments of the 1412 core genes obtained from the 221 Brazilian genomes (Fig. 1a). The 105 genomes from chicken carcasses that we sequenced were intermingled with the 116 genomes from the NCBI. MLST [40] revealed a total of 16 clonal complexes (CCs) and 54 STs that have been previously described (Table S1). The most common CCs were CC353 (consisting of 87/221 genomes or 39.37 % from ST353 and nine less common STs), CC607 (consisting of 25/221 genomes or 11. 31 % from ST607, ST6700 and three other less common STs), CC21 (consisting of 18/221 genomes or 8.14 % representing five STs) and 16/221 genomes or 7.24 % each of CC48 (representing three STs) and CC443 (representing three STs). Three novel STs were present in our dataset (ST13682, ST13683 and ST13684 assigned by pubMLST [41]). Three genomes have two new alleles in the gene *uncA* (ST13706 and ST13719), while two genomes have a new allele in the *tkt* gene (ST13721). There were 12 STs that contained six or more genomes, which altogether represented 67.42 % of the dataset (149/221 genomes). The most prevalent ST was ST353 (51/221 genomes or 23.07 % of the population) and consisted of samples from humans and chickens. ST353 was present throughout the last three decades and in all seven locations. Other frequently detected STs were as follows: ST8741 (15/221 genomes or 6.79 %) from chicken and human samples isolated between 2001 and 2018 in the states of MG, SP and RS; ST6700 (14/221 genomes or 6.33 %) included only samples sequenced in this study and is composed of chicken samples isolated between 2011 and 2018 in the states of PR, SC and RS; and ST475 (12/221 genomes or 5.43 %) from chicken, human and monkey sources isolated over three decades in PR, SC, RJ and SP.

The Brazilian *C. jejuni* population can be partitioned into 13 distinct SCs based on Bayesian hierarchical clustering of the core genome alignment (Fig. 1a, b). A total of four genomes could not be classified within any of the 13 clusters. The largest SC was SC9, which comprised 56 genomes (corresponding to 23.34 % of the population), of which 51 were ST353 genomes. SC9 was present in all the seven states in the study. SC5 consisted of 25 genomes (corresponding to 11.31 % of the population) from ST607 (8 genomes), ST6700 (14 genomes) and 3 genomes from less common STs. SC5 was present in five states. SC8 consisted of 20 samples (corresponding to 9.05 % of the population) and included ST475 (12 genomes) and 8 genomes from other less common STs. STs 353, 403 and 475 were consistently detected in all three decades, despite the differences in the number of genomes across time intervals (Fig. 1c).

Of the 12 STs that were represented by at least six genomes, STs 443, 1359, 6175, 2036 and 6700 consisted solely of chicken samples, most of which were isolated between 2011 and 2018. *C. jejuni* derived from human sources were present in ST52 (2 from human out of 6 genomes), ST353 (10 out of 51 genomes), ST403 (2 out of 8 genomes), ST463 (1 out of 6 genomes), ST475 (6 out of 12 genomes), ST607 (1 out of 8 genomes) and ST8741 (1 out of 15 genomes). We did not find any STs consisting entirely of human samples. Monkey-derived *C. jejuni* was identified in ST52 (4 from monkey out of 6 genomes), ST403 (4 out of 6 genomes) and ST475 (1 out of 12 genomes). Overall, these results show that the Brazilian *C. jejuni* population is phylogenetically diverse but has been dominated by ST353 (CC353; SC9) derived from human and chicken sources. This ST has persisted in the last three decades and is found in all seven states included in this study.

**Fig. 2.**
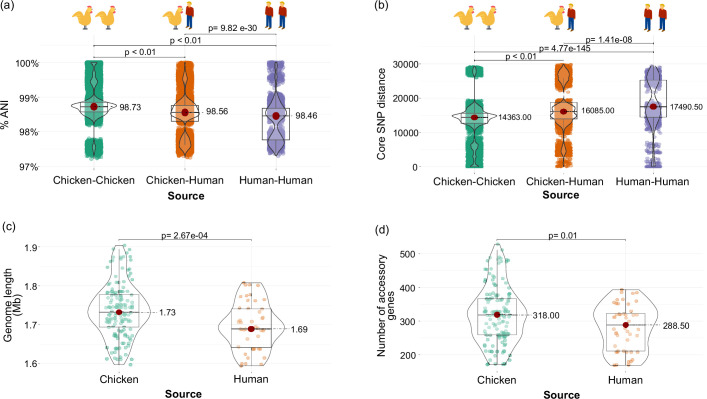
Genomic comparison of *C. jejuni* derived from chicken and human sources. (**a**) Genome-wide average nucleotide identity (ANI) values for all possible pairs of *C. jejuni* genomes within and between hosts. (**b**) Core genome distance calculated from SNP within and between hosts. In panels (a) and (b), coloured dots represent pairwise comparisons in each category. The Kruskal–Wallis test was used to compare paired groups in panels (a) and (b). Comparison of (**c**) genome length and (**d**) number of accessory genes between chicken- and human-derived *C. jejuni*. In panels (c) and (d), coloured dots represent individual genomes in each category. The Mann–Whitney test was used to compare paired groups in panels (c) and (d). In all panels, the mean value is represented by the red dot, the box represents the interquartile range, the horizontal line in the middle of the box represents the median and the lower and upper ends of the violin jitter plots represent the lowest data point without the outliers and the highest data point without outliers, respectively.

Our dataset consisted mostly of genomes recovered from chickens and humans. We therefore sought to compare the genomic features of *C. jejuni* from these two hosts. We obtained significant differences in pairwise genome-wide ANI values and core genome SNP distances when comparing genomes from within and between hosts (all *P*-values <0.01, Kruskal–Wallis test; Fig. 2a, b). We found significant differences between chicken- and human-derived *C. jejuni* genomes in terms of their genome length and number of accessory genes per genome (all *P*-values <0.01, Mann–Whitney test; Fig. 2c, d). The size of the *C. jejuni* genomes from chickens ranged from 1.60 to 1.90 Mb (mean=1.73 Mb), while those from humans ranged from 1.59 to 1.80 Mb (mean=1.69 Mb). The size difference between the two groups lies in part in their accessory genomes. Genomes of chicken-derived isolates carry more accessory genes (mean=318 genes; range=171–528) than those genomes from human sources (mean=288.5 genes; range=156–413). These results show that the host source influences the overall core and accessory genomic features of *C. jejuni*.

### Brazilian *C. jejuni* in the context of the South American *C. jejuni* population

To place the Brazilian *C. jejuni* in the broader South American population, we retrieved 374 genomes from the NCBI and combined them with the 221 Brazilian genomes. We built a maximum likelihood tree based on SNPs extracted from the nucleotide sequence alignments of 1377 core genes (Fig. S2). We found a diverse population composed of 130 STs belonging to 23 known CCs (Table S4), with Brazilian genomes intermingled within the phylogeny. CC353 was the most prevalent (135/595 genomes or 22.69 % of the population), with 64 ST353 genomes from chicken and human sources in Brazil, Chile, Ecuador and Peru. CC21 was the second most prevalent CC (78/595 genomes or 13.11 %) and was found in genomes from chicken and human sources in Brazil, Chile, Ecuador and Peru. Within CC21, genomes from Brazil and Chile clustered together in ST21 (humans), ST50 (humans) and ST1359 (humans and chickens). The third most prevalent CC was CC362 (72/595 genomes or 12.10 %), consisting of genomes from chickens and humans in Chile and Peru. This CC included ST2993, which was related to the 2019 GBS outbreak in Peru [20]. The fourth most prevalent was CC607 (42/595 genomes or 7.06 %) from humans and chickens in Brazil, Chile, Ecuador and Peru. Within CC607, ST607 encompassed genomes from chickens and humans in Brazil, Ecuador and Peru. Overall, many of the Brazilian genomes appear to be closely related to other lineages circulating in other parts of South America.

### Brazilian *C. jejuni* from different sources carries numerous virulence determinants

**Fig. 3.**
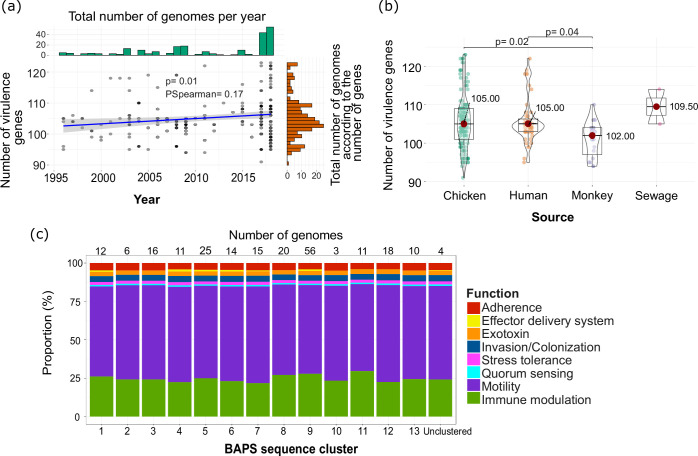
Distribution of virulence genes in *C. jejuni* from Brazil. (**a**) Relationship between the number of virulence genes per genome as a function of time (years). Each grey dot represents a *C. jejuni* genome. (**b**) Comparison of the number of virulence genes per genome according to ecological source. The Kruskal–Wallis test was used to compare paired groups in panel (b). The mean value is represented by the red dot, the box represents the interquartile range, the horizontal line in the middle of the box represents the median and the lower and upper ends of the violin jitter plots represent the lowest data point without the outliers and the highest data point without outliers, respectively. (**c**) Distribution of virulence genes classified according to gene function among the different BAPS clusters.

We next sought to characterize the set of virulence genes carried by the Brazilian *C. jejuni* population. We identified a total of 139 unique virulence genes in the entire population (Table S5 and Fig. S2). We found a slightly positive but significant increase in the number of virulence genes per genome from 1996 and 2018 (Spearman’s correlation=0.17, *P*=0.01; Fig. 3a). However, we note that this is likely due to the higher number of genomes recovered from more recent years. Overall, genomes carried a range of 91–123 virulence genes per genome, with 25 genomes carrying 102 virulence genes per genome. In terms of ecological sources, we found that chicken- and human-derived *C. jejuni* did not vary in the number of virulence genes per genome with both groups carrying a mean of 105 genes per genome (Fig. 3b).

A variety of genes that contribute to targeting host cells, adhesion, colonization, invasion and survival inside the host were detected in all isolates, regardless of the host source [3,58]. The virulence genes we detected can be classified into eight major functions (Figs. 3c and S3). The most prominent were those related to motility, of which we identified 66 unique motility-associated genes in the entire population. A total of 51 motility-related genes were identified in all genomes. Two of the most frequently detected motility-related genes were *flaA* and *flaB* genes, which encode the two flagellins that are important for motility, secretion of the Cia proteins and adhesion to biotic and abiotic surfaces in *C. jejuni* [59,60]. These were present in 29.41 % (65/221 genomes) and 28.95 % (64/221 genomes) of the population, respectively, and were detected only in chicken- and human-derived *C. jejuni* in all SCs except SC1 and SC10. The *flgE2* gene, which encodes a flagellar hook protein and is required for motility, flagellar assembly and protein secretion [59,60], was present in 49.96 % of the population (106/221 genomes) in all SCs, except SC1, SC4, SC8 and SC12. The *pseD* gene is a motility accessory factor that functions in flagellin glycosylation and was present in 44.80 % (99/221 genomes) of the population in all SCs, except SC13. We identified 52 genes associated with immunomodulation, of which 14 genes were detected in all genomes (Table S5 and Fig. S2): *gmhAB*, *hldDE*, *htrB*, *kpsD*, *kpsEFMST* and *waaCFV*. We detected virulence genes related to exotoxins (*cdtABC*), invasion and colonization (*ciaCB*, *pldA* and *htrA*), stress tolerance (*dnaJ*, *sodB*), quorum sensing (*luxS*) and adherence (*Cj1279c*, *cadF* and *pebA*) in all genomes.

**Fig. 4.**
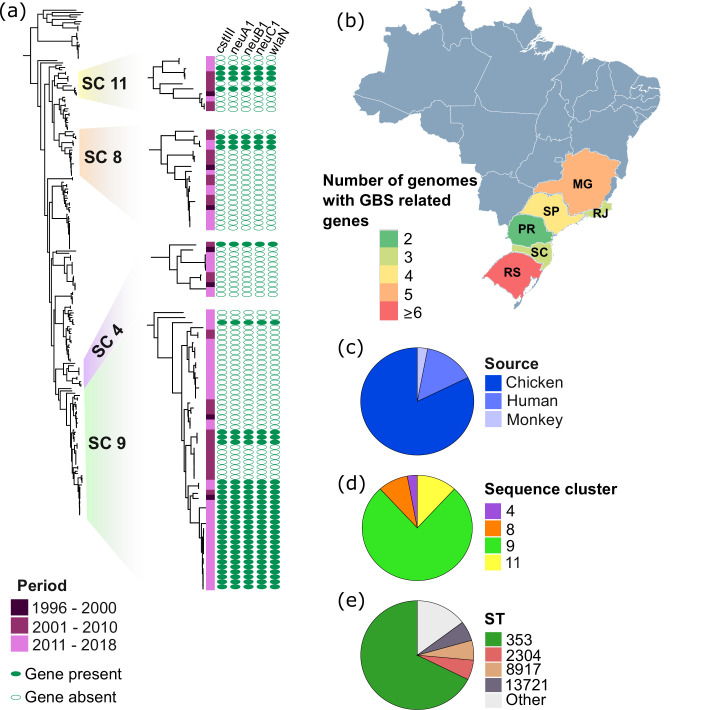
Presence and distribution of genes *cstIII*, *neuA1*, *neuB1*, *neuC1* and *wlaN*, associated with the development of Guillain–Barré syndrome (GBS). (**a**) Phylogenetic tree showing the four BAPS clusters SC4, SC8, SC9 and SC11, which contain genomes carrying GBS-associated genes. Filled ovals indicate the presence of the gene. Distribution of genomes carrying GBS-associated genes according to (**b**) state, (**c**) source, (**d**) sequence cluster and (**e**) ST.

Notably, we identified 34 genomes carrying five genes associated with the induction of the rare autoimmune disorder GBS (*cstIII*, *neuA*, *neuB*, *neuC* and *wlaN*; Fig. 4a) [61,62]. Genomes harbouring GBS genes were present in all states except DF, with 17 out of 34 genomes from RS (Fig. 4b). Out of the 34 genomes carrying GBS genes, 28 were derived from chickens (Fig. 4c). The GBS genes were detected in four sequence clusters SC4, SC8, SC9 and SC11, with the majority coming from SC9 (26 out of 34 genomes) (Fig. 4d). Most of ST353 (23 out of 34 genomes; CC353; SC9) contain the GBS genes (Fig. 4e). Other STs that contain genomes with the GBS genes included ST2304 (chicken), ST8917 (chicken), ST13721 (chicken), ST50 (human), ST1522 (human), ST6091 (monkey), ST9083 (human) and ST12542 (chicken). We did not find isolates belonging to well-known GBS-associated STs and CC, notably ST22 [63].

### AMR determinants are diverse and widespread in Brazilian *C. jejuni* from different sources

**Fig. 5. F5:**
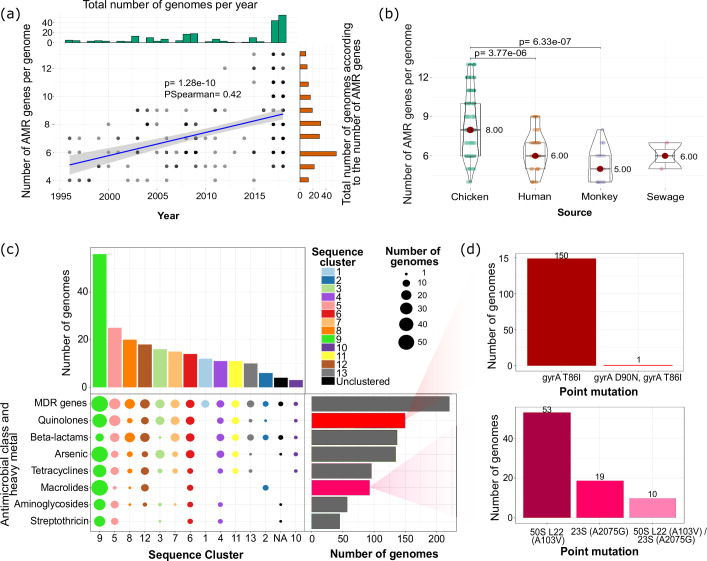
Antimicrobial resistance (AMR) in Brazilian *C. jejuni* isolates. (**a**) Relationship between the number of AMR genes per genome as a function of time (years). Each grey dot represents a *C. jejuni* genome. (**b**) Comparison of the number of AMR genes per genome according to the ecological source. The Kruskal–Wallis test was used to compare paired groups in panel (b). The mean value is represented by the red dot, the box represents the interquartile range, the horizontal line in the middle of the box represents the median and the lower and upper ends of the violin jitter plots represent the lowest data point without the outliers and the highest data point without outliers, respectively. (**c**) Distribution of genomes carrying at least one gene or mutation conferring resistance to each antimicrobial class and heavy metal. The coloured circles correspond to the sequence cluster from which the genomes with resistance genes belong, and the size of the circle is proportional to the number of genomes. MDR genes refer to multidrug resistance genes, which were the genes related to the *cmeABCR* encoding the multidrug efflux pump. NA refers to the unclustered genomes in panel (c)[Fig F5]. (**d**) Number of genomes carrying specific mutations related to resistance against quinolones and macrolides.

We next sought to determine the composition and distribution of AMR and heavy metal resistance genes in the *C. jejuni* population in Brazil. We identified a total of 24 unique AMR genes and 2 arsenic resistance genes in our dataset (Table S5 and Fig. S4). A significant increase in the number of AMR genes per genome can be observed throughout the period of our study (Spearman’s correlation=0.42, *P*=1.28e-10; Fig. 5a). Overall, genomes carried a range of 4–13 AMR genes per genome. Chicken-derived * C. jejuni* harboured a higher number of AMR genes per genome compared to human-derived genomes (mean of 8 and 6, respectively; Kruskal–Wallis test, *P*=3.77e-06) (Fig. 5b).

Sequence cluster SC9 consisting mostly of ST353 carried the highest number of AMR determinants per genome (range = 6–13, mean=9). Sequence clusters SC5, SC6 and SC9, which were mainly composed of *C. jejuni* isolated from chickens, contained genomes that carried at least one resistance gene related to each antimicrobial class or heavy metal (Fig. 5c). A total of 56 SC9 genomes contained at least one gene or mutation conferring resistance against quinolone, beta-lactam, tetracycline, macrolide, aminoglycoside and streptothricin. Notably, all SCs included genomes that harboured the operon *cmeABCR* related to the multidrug efflux pump, which contributes to resistance against tetracyclines, macrolides and quinolones [64].

A variety of AMR genes were differentially distributed among the SCs of *C. jejuni* [8]. We detected a variety of genetic determinants that confer beta-lactam resistance that include *bla* genes from the OXA-61 family (*blaOXA*, *blaOXA-193*, *blaOXA-449*, *blaOXA-450*, *blaOXA-460*, *blaOXA-461*, *bla-OXA489*, *bla-OXA595* and *blaOXA-603*) and OXA-184 family (*blaOXA-184*) [65] (Table S5). A total of 138 genomes or 62.44 % of the entire dataset contained at least one of these genes. The most prevalent *bla* genes were *blaOXA-193* (present in 79 genomes or 35.75 % of the dataset) and *blaOXA-184* gene (present in 23 genomes or 10.40 %). Tetracycline resistance is conferred by the presence of the *tetO* gene [66] or the mosaic *tetO/M/O* gene [67]. A total of 96 genomes carried at least one of these tetracycline resistance genes. Resistance to aminoglycosides was identified by the presence of the genes *aad9*, *aadE* and *aph(3')-IIIa* [68]. A total of 57 genomes or 25.79 % of the dataset contained at least one of these genes. Streptothricin resistance conferred by the *sat4* gene [69] was detected in 45 genomes. Lastly, we identified 135 genomes carrying *arsP* gene and 45 genomes carrying *acr3* conferring resistance against arsenic [70].

We detected mutations related to resistance against quinolones and macrolides (Fig. 5d). These include two quinolone-related point mutations in the *gyrA* gene encoding the DNA gyrase A enzyme [71]. The *gyrA* T86I mutation was present in 150 genomes (or 67.87 % of the dataset). We detected one genome from a human source in the sequence cluster SC13 that contained two quinolone-related mutations in the *gyrA* gene (T86I and D90N). We detected the presence of two mutations in ribosomal genes related to macrolide resistance [72]. A total of 53 and 19 genomes contained the 50S rRNA L22 (A103V) and the 23S rRNA (A2075G) mutations, respectively. There were ten genomes that carried both mutations. Genomes carrying the 23S (A2075G) mutation were derived only from chicken. In contrast, genomes positive for the 50S L22 (A103V) mutation were obtained from chickens, humans and monkeys. While the presence of 50S L22 (A103V) point mutation alone does not induce phenotypic resistance to azithromycin and erythromycin, synergy with the CmeABC efflux pump is capable of conferring resistance to these drugs [73].

### Temporal origins and population dynamics of ST353 in Brazil

**Fig. 6.**
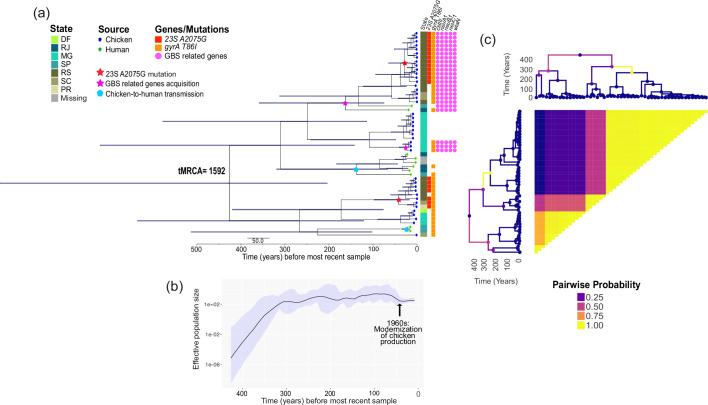
Time-calibrated phylogeny and changes in effective population size of ST353 in Brazil. (a) Bayesian maximum clade credibility time-calibrated phylogeny estimated by BactDating and built from the non-recombining regions of the core genome alignment. The time to the most recent common ancestor (tMRCA) is shown on the most ancestral node. Blue horizontal bars at each node represent 95 % confidence intervals. The presence or absence of relevant genes or mutations is shown on the matrix on the right of the tree. (b) Bayesian skygrowth plot showing the change in effective population size (*Ne*) over time. The median is represented by the black line and 95 % confidence intervals are in blue shade surrounding the median values. (c) Dated phylogeny with branches coloured according to the inferred probability of clonal expansion estimated by CaveDive. Pairwise matrix showing the posterior probabilities of any two genomes belonging to the same subpopulation.

We next sought to determine the temporal origins of the high-frequency lineage ST353 in Brazil. Using BactDating [46], we observed a positive correlation between isolation date and root-to-tip distance for this ST (*R*^2^=0.43 and *P*<1e-04; Fig. S5a), indicating the presence of a clock-like signal. We, therefore, carried out a dated coalescent phylogenetic analysis and estimated that the tMRCA of ST353 to be 1592, although we recognize that the 95 % highest posterior density (HPD) intervals were large and thus should be considered with caution (1050–1820; Fig. 6a). For verification, we used the coda package [50] to analyse convergence and mixing (Fig. S5bc) and ensured that the effective population size was greater than 200 and the Gelman–Rubin statistic less than 1.1 for all variables (Table S7).

Ancestral branches in the time-calibrated phylogeny represented chicken-derived isolates and thus were likely the earliest host of ST353. Two ancestral chicken-to-human infection events likely occurred, and the estimated dates of these infections were 1879 (HPD=1699–1950) and 1996 (HPD=1984–2003). It is also likely that a third chicken-to-human infection occurred in 1854 (HPD=1658–1948) and subsequent human-to-chicken infections occurred in 1885 (HPD=1706–1958). Equally possible is the scenario whereby two contemporary human-derived isolates (SRR6314746 in 1999 and SRR6314024 in 2006) independently experienced chicken-to-human infections. It is important to note that the true mode of transmission may not be accurately inferred due to incomplete sampling and missing intermediate hosts.

Our time-calibrated phylogenetic analysis allowed us to infer the times that certain AMR and virulence determinants appeared in ST353. Two subclusters within ST353 contain the five genes associated with GBS, and they appeared in 1854 (HPD=1658–1948) and 1992 (HPD=1978–2002). The A2075G mutation on the gene that encodes the 23S rRNA conferring macrolide resistance appeared in 1976 (HPD=1925–2003) and 1990 (HPD=1953–2017) independently in two separate subclusters. One of these two clusters already harboured the GBS genes, indicating convergence of AMR and virulence determinants in an ancestral strain of ST353. The T86I mutation on the *gyrA* gene associated with resistance to quinolone [71] appeared in the tMRCA of ST353 or alternatively, at least four times throughout the history of ST353. Altogether, multi-host infections were accompanied by the acquisition of different gene combinations of AMR and virulence determinants.

We estimated the change in the effective population size (*Ne*) of ST353 (Fig. 6b). Population demographic analysis shows that the *Ne* rapidly increased from the late 1500s to the late 1600s, coinciding with the time of the early settlement of the Portuguese first arrived in Brazil in 1500 [74]. Beginning in the early 1700s, *Ne* plateaued, which coincided with the start of the mining period in Brazil in 1693 [75]. This stable population growth continued until 1968 when a slight decline in *Ne* was observed. The time of this decline coincided with the time of the modernization of large-scale chicken production in the country for global export and consumption [16,17]. However, time-scale estimates of ancestry are affected by the sample size, limitations of the evolutionary models used and niche segregation of the bacterial species, and hence, we can only speculate on these temporal estimates [76].

Lastly, we sought to identify whether there is a signal indicating a clonal expansion in ST353 in Brazil (Fig. 6c). We used CaveDive [53] and evaluated the results using the coda package [50] to ensure that the effective sample sizes for all parameters were greater than 200 and the Gelman–Rubin statistic less than 1.1 for all variables (Table S8). Examining the entire Brazilian ST353 dataset, we found posterior probability estimates of 60.4 %, 35.5 % and 3.8 % for having zero, one and two clonal expansions, respectively. The most probable posterior population structure therefore consists of zero expansion, although the possibility of one expansion cannot be completely ruled out, as CaveDive tends to underestimate the number of clonal expansions [53]. However, although our analysis showed that even with the overall posterior probability of zero clonal expansion (Fig. S6ab), a sublineage within ST353 (branch coloured in yellow in Figs. 6c and S6cd) revealed a possible clonal expansion that likely occurred 250 years ago. Interestingly, the time of the clonal expansion of this sublineage coincided with the time when GBS-related genes were estimated to be acquired.

## Discussion

Understanding the population genomic structure of the foodborne pathogen *C. jejuni* is critical to informing public health interventions and infection control policies. Our analysis of 221 *C*. *jejuni* genomes from Brazil revealed the growing importance of ST353 (SC9) from chickens in shaping the dynamics of human infection, transmission and spread of AMR. Our study highlights the need for continued surveillance of *C. jejuni* in clinical and agricultural settings to control its spread and limit its impact on food security and public health.

Surveillance studies of *Campylobacter* in Brazil using whole-genome sequencing have been few [77,79]. Hence, we have an incomplete picture of how the aetiologic agent is changing and evolving as well as the drivers that contribute to campylobacteriosis in the country. The convergence of multidrug resistance and virulence determinants in certain lineages, as in the case of ST353 (SC9), should be considered a priority in surveillance efforts. Through different routes, *C. jejuni* is constantly exposed to antimicrobials widely used for food production. The presence of a wide range of resistance determinants against antimicrobial compounds and heavy metals in *C. jejuni* from both chicken and human sources is a cause for concern. *C. jejuni* is known to harbour mobile genetic elements that carry AMR genes, thus facilitating their dissemination between hosts and environments [8,80]. Horizontally transferable AMR genes and the high mutability in genes associated with resistance to quinolones and macrolides can lead to the persistence and adaptation of high-risk clones in food-producing environments where antimicrobials are frequently used. The high frequency and widespread distribution of ST353 can be attributed in part to the numerous mechanisms of resistance it harbours that allow it to overcome selective pressures in clinics and farms.

While ST353 is considered a chicken specialist [81,83], its presence in human sources means that chicken-to-human transmission may have been overlooked in the country. It is possible that ST353 outbreaks have occurred in Brazil, but the lack of sustained surveillance may not have identified such cases. ST353 (or CC353 in general) is a globally important lineage and has been reported in Poland [84], the Baltic States [85], Israel [86], Nigeria [87], China [88], the USA and the UK [14]. Future genomic investigations will be critical to documenting whether ST353 will continue to persist in Brazil or less common STs will replace it over the long term. While not as widespread as ST353, STs 607/6700 (sequence cluster SC5), STs 443/463 (sequence cluster SC12) and ST 8741 (sequence cluster SC3) appear to have the potential to become more prevalent in the population. Nonetheless, even if ST353 continues to dominate the population in future years, less common STs can act as important sources of horizontally mobile DNA.

The presence of genes associated with the autoimmune peripheral neuropathy GBS in the Brazilian *C. jejuni* across host sources, SCs and STs, was particularly intriguing. Our results are consistent with previous reports of GBS genes in animals in other parts of the world. In the USA, *C. jejuni* from chicken and cattle across the food production chain, encompassing live food animals, poultry processing and retail meat, revealed the presence of the GBS virulence genes *neuABC* and *cstIII* in 57.6 and 26.8 % of the isolates across multiple STs, respectively [89]. In Switzerland, a total of 37 % of the *C. jejuni* isolates, mainly from ST21 and ST50, from broiler carcasses co-harboured *cstIII*, *neuA1*, *neuB1* and *neuC1*, while 8 % carried the *wlaN* gene [90]. These broiler carcasses came from animals from three different types of farming systems: animal-friendly stabling, free-range farms and organic farms [90]. Similar results have been reported in *C. jejuni* from caeca and carcass neck skin sampled at three poultry slaughterhouses in Ireland [91]. These previous studies as well as our analysis suggest that the *C. jejuni* strains found in different host sources have the potential to cause GBS, with little selection by the host. The non-host-specific distribution of GBS genes has important implications in multi-host transmission and in the development of neuropathy in animals. In the 2019 outbreak in Peru, chickens are the likely reservoir of GBS-related *C. jejuni* ST2933 [20]. Occupational contact with live poultry or swine was associated with increased reporting of GBS-like symptoms, mainly numbness and weakness [92], with higher anti-*C. jejuni* antibodies in animal production farmers with past or present animal exposure [93]. Neuropathic sequelae developed in dogs that consumed raw poultry meat [94] and in chicken after experimental inoculation with *C. jejuni* isolated from GBS patients [95,96]. However, we note that our dataset is limited as there were no isolates belonging to well-known GBS-associated STs and CC, notably ST22 [63]. Nonetheless, it is important to assess the risks of developing symptoms consistent with GBS among farmers and animal production workers, consumers and animals.

It is important to understand the historical context in which *C. jejuni* has evolved in Brazil. In 1500, the Portuguese introduced domesticated chickens in the country mainly for local consumption [16,17]. The early poultry farming and the migration of the Portuguese and their livestock may have opened new ecological niches for the pathogenic lineages that accompanied the trans-Atlantic transport of chickens and/or for the endemic *C. jejuni* lineages already circulating in Brazil. In 1693, the mining period started in Brazil [75], which led to a massive population growth of Brazilians [75,97]. In the 1960s, Brazil implemented an industrialized approach to poultry farming that transformed Brazil to become one of the largest chicken producers and exporters today [16,17]. Industrialization can lead to an expanding niche for prevailing pathogenic lineages and more opportunities for the emergence of more adaptive lineages. Intensification of chicken production may have enabled the appearance of different sets of certain AMR and virulence determinants in ST353 at multiple times throughout its history in Brazil. For example, resistance to macrolides via the 23S rRNA A2075G mutation appeared in 1974 and 1989 in two sublineages of ST353. This may be related to the use of some macrolides as growth promoters, a practice that began in the 1960s [98] and has only recently been banned from poultry production in Brazil. Erythromycin and spiramycin were banned in 2012, and tylosin was the last to be banned in 2020 [99]. Our results parallel recent findings on the impact of agricultural intensification in other bacterial pathogens. The ST61 cattle specialist of *C. jejuni* emerged as a result of the dramatic rise in the number of cattle worldwide during the 20th century [100]. The emergence and global spread of bovine-associated pathogenic lineages of *Streptococcus suis* in the 19th and 20th centuries occurred during an early period of growth in pig farming [101]. The global expansion of *Staphylococcus aureus* clones causing bovine mastitis can be traced to the mid-19th to late 20th century coinciding with the commercialization and industrialization of dairy farming [102].

Our study is not without limitations. We acknowledge that several states in Brazil and other animal hosts were not represented in our dataset. *C. jejuni* is known to colonize a variety of livestock, companion and wildlife species [5] and can survive in water and soil [103]. Its ability to adapt to a wide range of environments therefore means that there are important reservoirs of infection and routes of transmission that needed to be further investigated. Surveillance of ST353 in Brazil as well as in other countries in South America may uncover other instances of multi-host infection, unique sets of AMR determinants, other virulence genes or unrecognized reservoirs. It is possible that ST353 may be more prevalent only in the southeastern part of Brazil, and hence, sampling in other regions may reveal other more prevalent STs. We also do not have data on *in vitro* antimicrobial susceptibility testing of the Brazilian isolates we sequenced. Nonetheless, our study should be considered as a baseline census of the standing genomic and lineage diversity of *C. jejuni* in the country. Our findings will inform current epidemiological efforts and create an impetus to initiate a wider and more systematic sampling scheme in both clinical and agricultural settings.

Our study reveals that the evolution of *C. jejuni* in Brazil was driven by the convergence of clinically relevant genetic elements, multi-host adaptation and clonal population growth partly attributed to major socio-economic changes in poultry farming. Restricting the spread of pathogenic and resistant lineages of *C. jejuni* in poultry populations should therefore be a priority to limit its impact on food security and public health. Pathogen genome sequencing across different One Health [104] sectors, especially in poorly surveyed countries, will be instrumental in developing effective public health measures. In particular, our findings will inform efforts to reduce the burden of campylobacteriosis, including the use of effective antimicrobials, control of zoonosis, design of food safety measures, source traceback and outbreak detection.

## supplementary material

10.1099/mgen.0.001274 Uncited Supplementary Material 1.

10.1099/mgen.0.001274 Uncited Supplementary Material 2.

## Abbreviations

AMR: antimicrobial resistance
ANI: average nucleotide identity
GBS: Guillain-Barré syndrome
MLST: multi-locus sequence typing
SC: sequence cluster
ST: sequence type
tMRCA: time to the most recent common ancestor
