# Supplementary material for: Five centuries of genome evolution and multi-host adaptation of Campylobacter jejuni in Brazil
Source: Microb Genom. 2024 Jul 19;10(7):001274. doi: 10.1099/mgen.0.001274 (PMC11316555; doi:10.1099/mgen.0.001274)
Supplement: Uncited Supplementary Material 1. [file mgen-10-01274-s001.pdf]

Five centuries of genome evolution and multi-host adaptation of *Campylobacter jejuni* in Brazil

Ana Beatriz Garcez Buiatte[1,2], Stephanie S. R. Souza[2], Leticia Roberta Martins Costa[1], Phelipe Augusto Borba Martins Peres[3], Roberta Torres de Melo[1], Simone Sommerfeld[4], Belchiolina Beatriz Fonseca[4], Nicole I. Zac Soligno[2], Odion O. Ikhimiukor[2], Paulo Marcel Armendaris[5], Cheryl P. Andam[2#], Daise Aparecida Rossi[1#]

Supplementary information

Supplementary Table S1. Accession numbers, associated metadata, sequence quality metrics, sequence types (ST), clonal complexes (CC) and sequence clusters inferred by fastBAPS (fast Bayesian Analysis of Population Structure) of the 221 *C. jejuni* genomes in this study. New ST assignments are highlighted in yellow.

Supplementary Table S2. Genome-wide average nucleotide identity values (ANI) for all possible pairs of the 221 *C. jejuni* genomes.

Supplementary Table S3. List of all genes detected using Panaroo in the pan-genome of 221 *C. jejuni.* 1 and 0 represent the presence and absence of the gene in a genome, respectively. The genes are color-coded according to core (green), soft core (orange), shell (yellow), and cloud (red) categories.

Supplementary Table S4. Accession numbers, associated metadata, sequence quality metrics, clonal complexes (CC) sequence types (ST) of the 595 *C. jejuni* genomes from South America.

Supplementary Table S5. Distribution of acquired genes and mutations associated with antimicrobial resistance (AMR) and virulence genes for each *C. jejuni* genome. 1 and 0 represent the presence and absence of the gene (or mutation) in a genome, respectively.

Supplementary Table S6. Accession numbers of the genes used to construct the custom database to detect the virulence genes *sodB, dnaJ, luxS, hcp, pldA* and *htrA* using ABRicate.

Supplementary Table S7. Quality control check of Bactdating run for dated phylogeny of *C. jejuni* ST353 genomes in Brazil

Supplementary Table S8. Quality control check of CaveDive run on the dated phylogenetic tree of *C. jejuni* ST353 genomes in Brazil

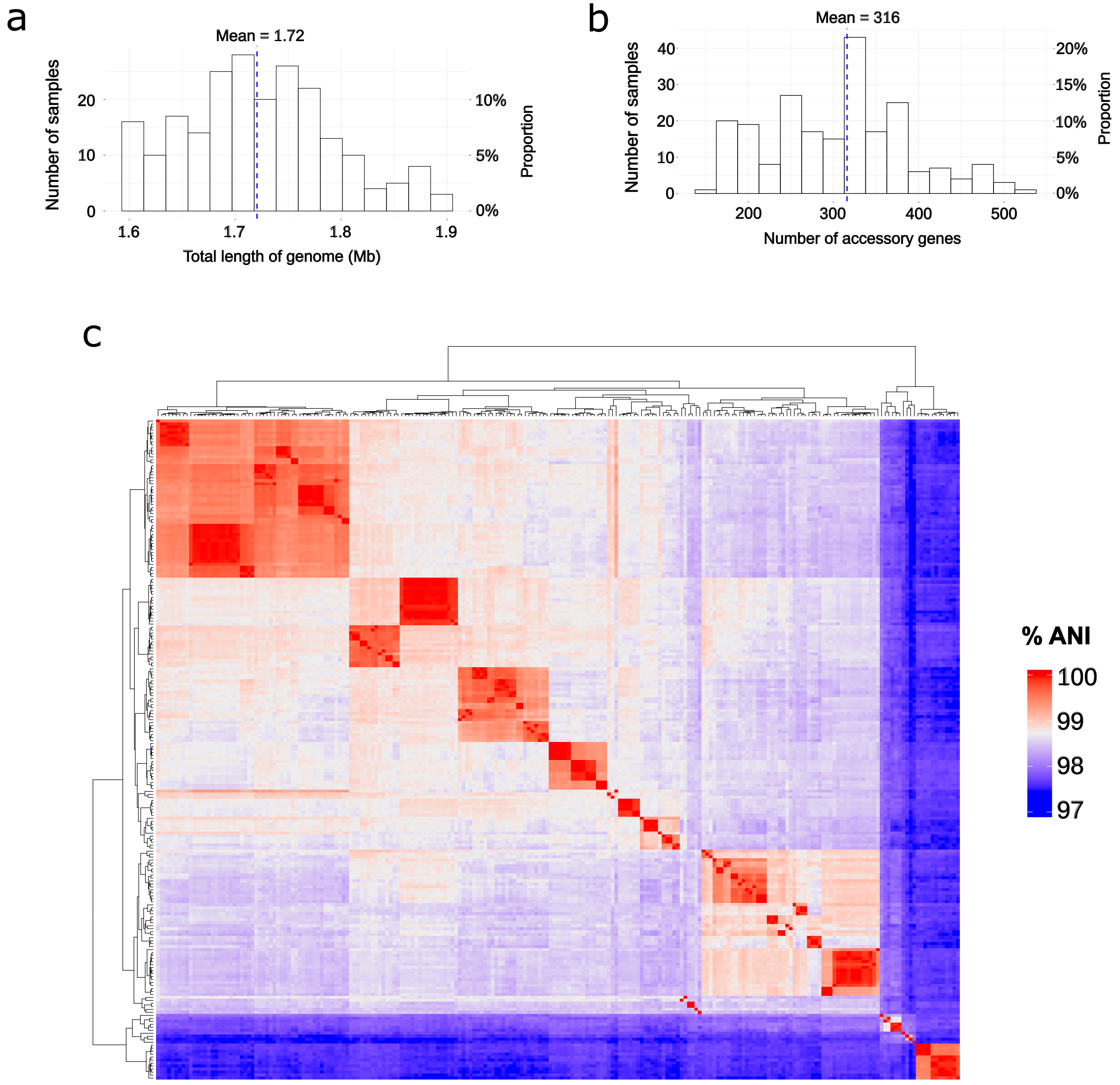

Supplementary Figure S1. Genomic features of the 221 *C. jejuni* genomes. (a) Genome length. (b) Number of accessory genes per genome. (c) Pairwise ANI values.

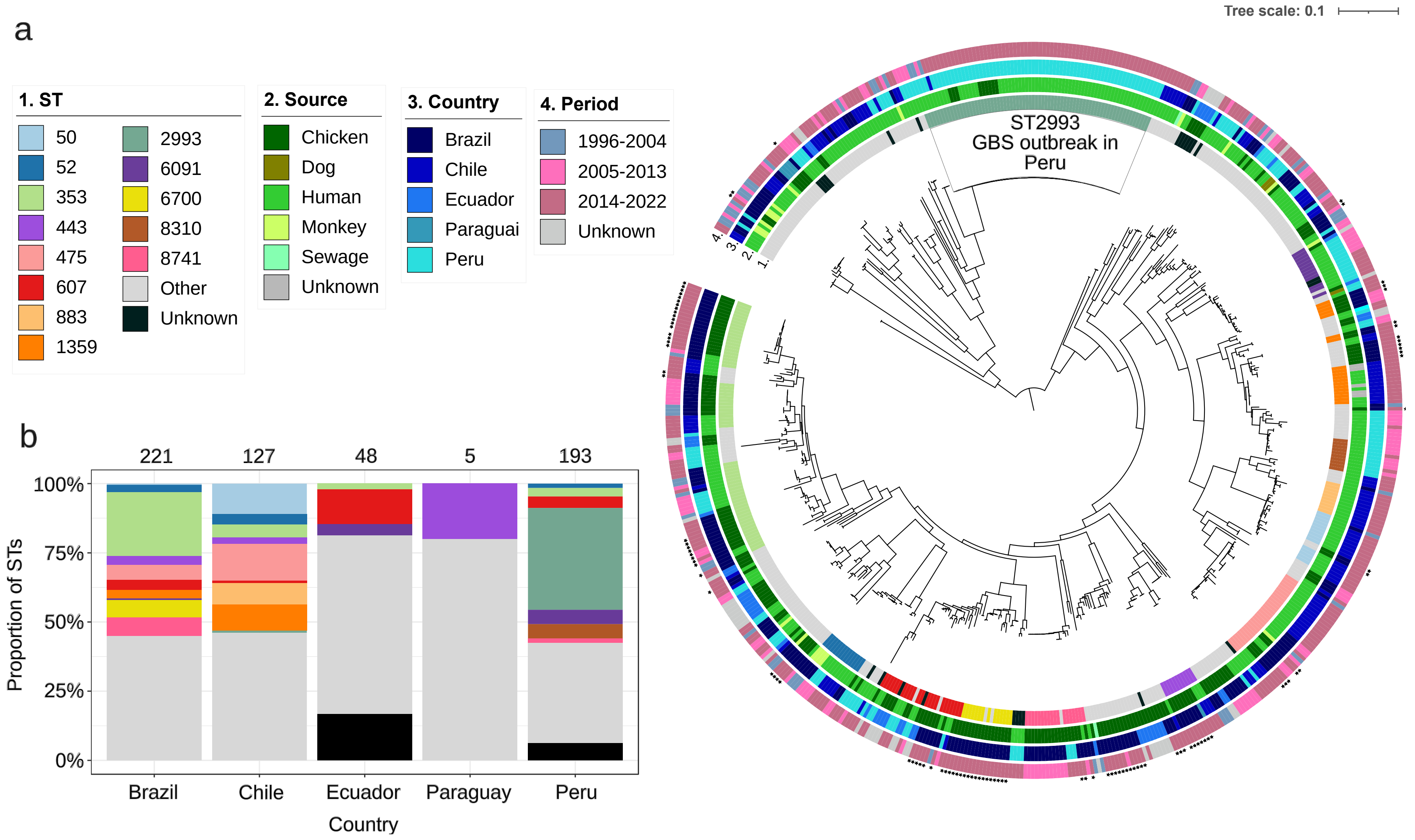

Supplementary Figure S2. (a) Midpoint-rooted maximum likelihood phylogenetic tree built from the sequence alignment of 1,377 core genes from 595 South American *C. jejuni* genomes. Tree scale represents the number of nucleotide substitutions per site. The black stars indicate the genomes that were sequenced in this study. Outer rings show the (1) ST, (2) source of isolation, (3) sampling site, and (4) year of sampling. (b) Distribution of STs in each country.

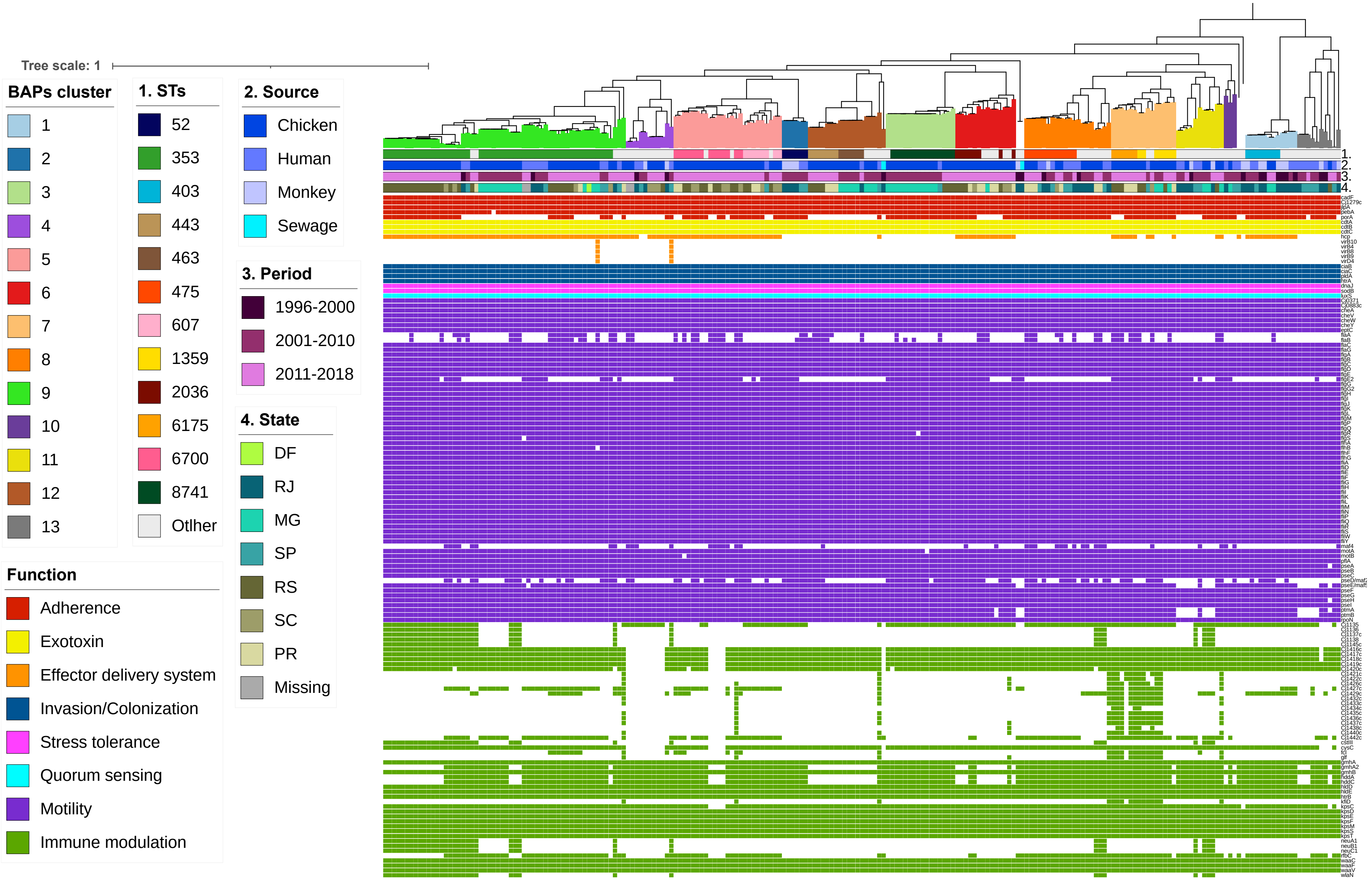

Supplementary Figure S3. Phylogenetic distribution of virulence genes in 221 *C. jejuni* genomes. The tree is identical to that in Figure 1a. Each column corresponds to a genome in the tree. Colored blocks indicate the presence of the gene. The same information can be found in Supplementary Table S4.

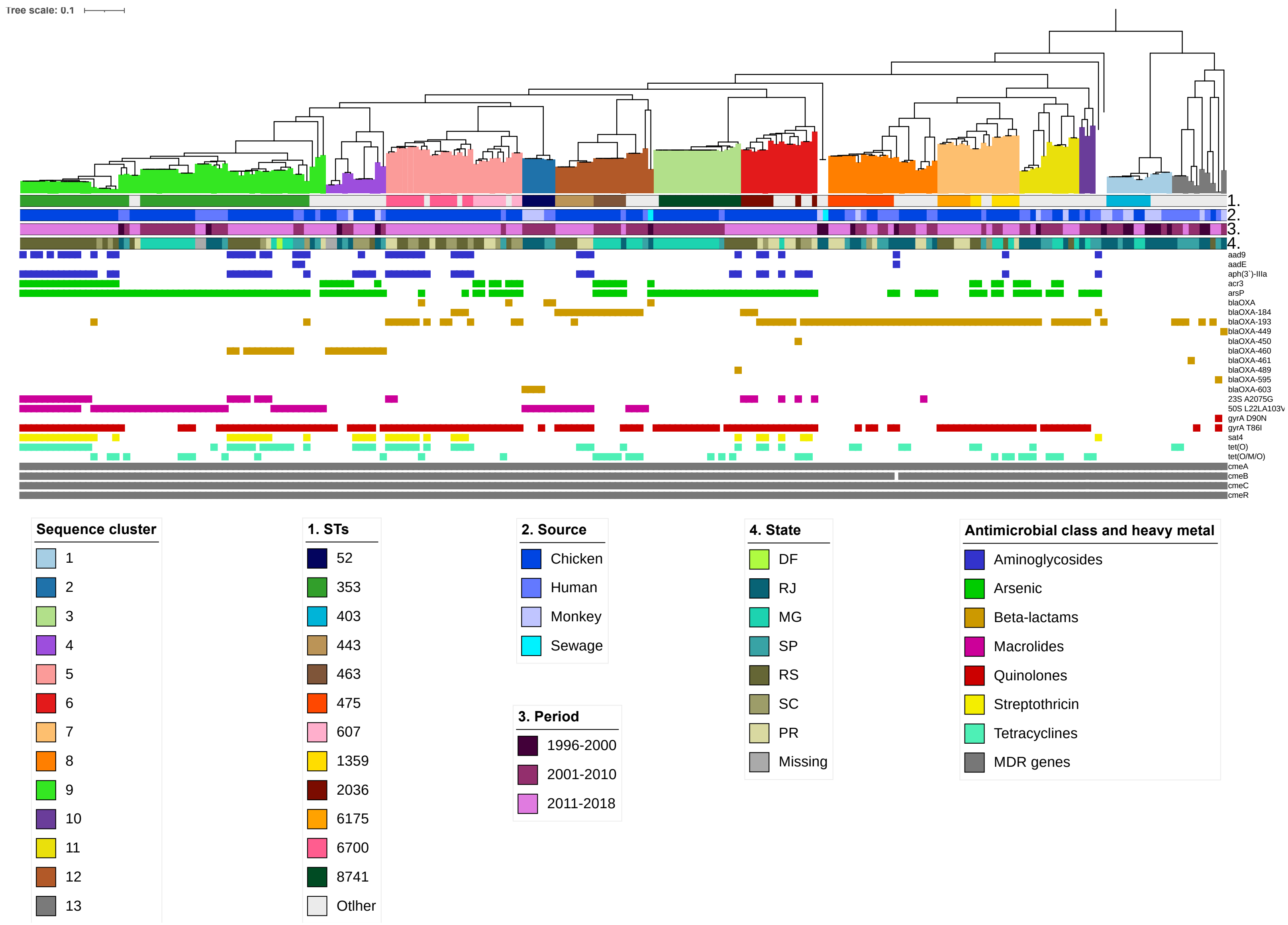

Supplementary Figure S4. Phylogenetic distribution of AMR and heavy metal resistance genes in 221 *C. jejuni* genomes. The tree is identical to that in Figure 1a. Each column corresponds to a genome in the tree. Colored blocks indicate the presence of the gene. The same information can be found in Supplementary Table S4

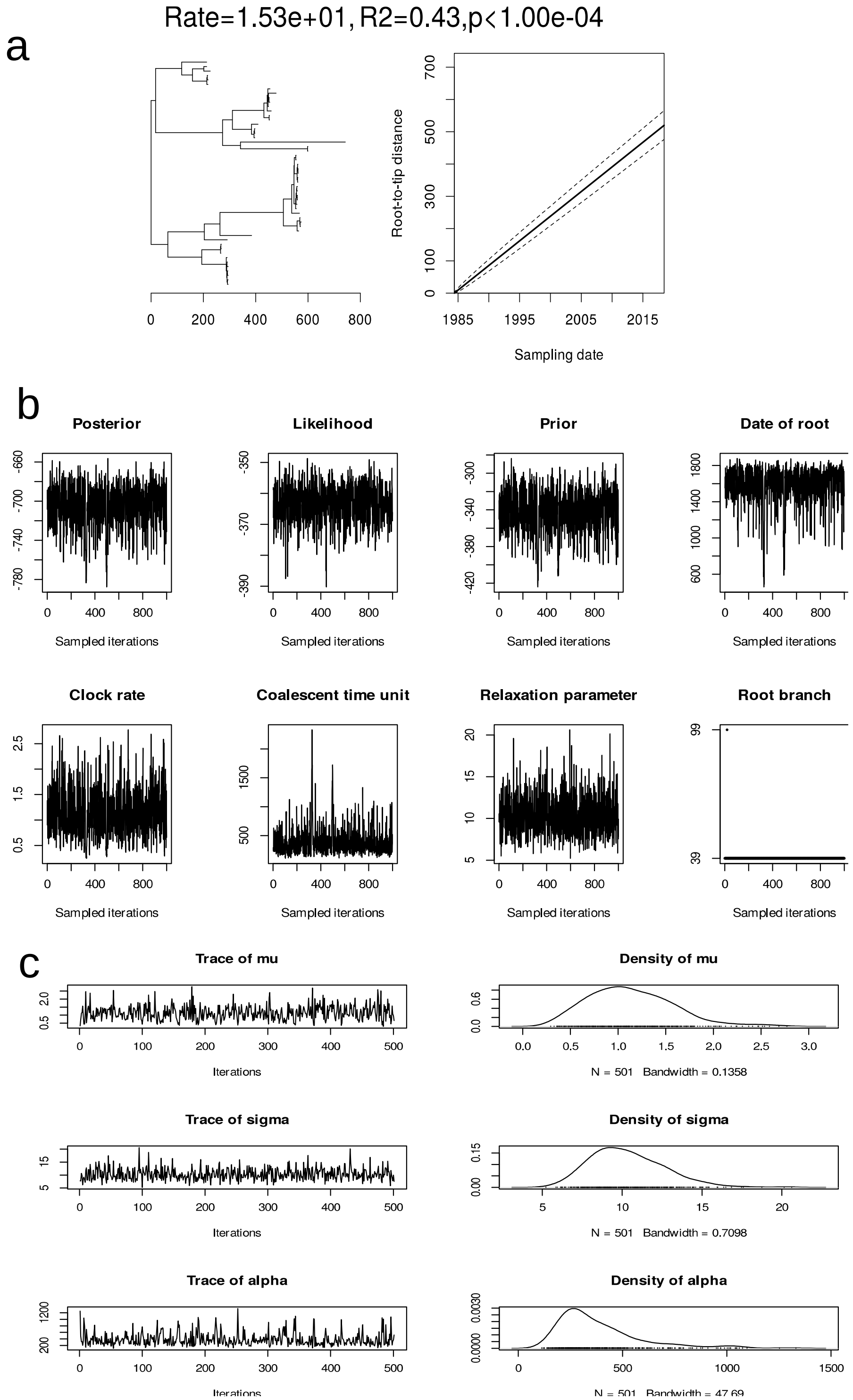

Supplementary Figure S5. Bactdating statistical tests and MCMC trace plots for ST353 in Brazil. (a) Initial rooted phylogeny and correlation test between date and root-to-tip distance. Dots are colored according to year of sampling of each isolate. (b). Bactdating trace plots constructed by periodic sampling over the MCMC runs. (c) Trace and density plots of mu, sigma and alpha.

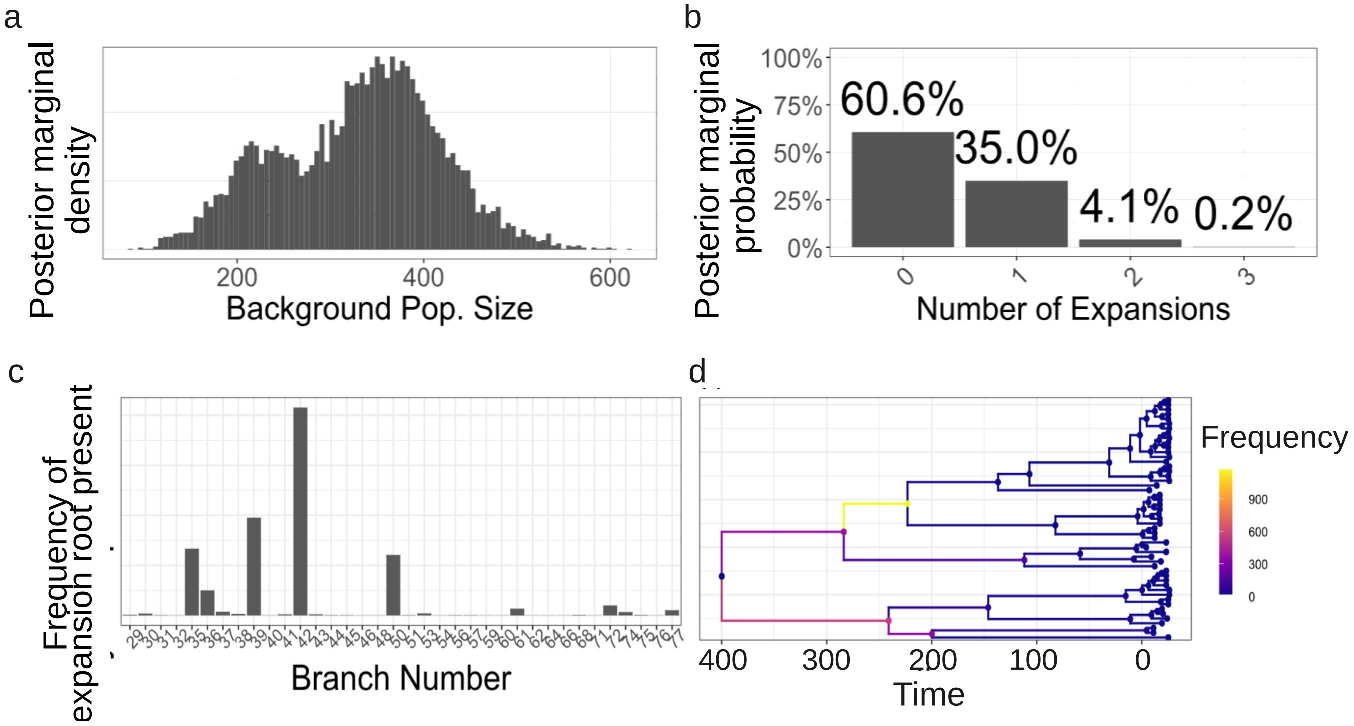

Supplementary Figure S6. CaveDive summary for ST353 in Brazil. (a) Posterior distribution of the background population size. (b) Posterior distribution of the number of clonal expansions. (c,d) Posterior probabilities of having a clonal expansions on different branches of the tree.
